# Insights into Microbiota in Sjögren’s Syndrome

**DOI:** 10.3390/medicina59091661

**Published:** 2023-09-14

**Authors:** Diana Mieliauskaitė, Vilius Kontenis

**Affiliations:** State Research Institute Center for Innovative Medicine, Department of Experimental, Preventive and Clinical Medicine, Santariskių St. 5, LT-08405 Vilnius, Lithuania; vilius.kontenis@imcentras.lt

**Keywords:** Sjögren’s syndrome, autoimmune diseases, microbiota, dysbiosis, molecular mimicry, metabolite, epithelial tolerance

## Abstract

Primary Sjögren’s syndrome (pSS) is a heterogeneous chronic autoimmune disorder with multiple clinical manifestations that can develop into non-Hodgkin’s lymphoma in mucosa-associated lymphoid tissue. The pathogenesis of Sjögren’s syndrome (SS) is not completely understood, but it is assumed that pathogenesis of SS is multifactorial. The microbiota plays a notable role in the development of autoimmune disorders, including Sjögren’s syndrome. Molecular mimicry, metabolite changes and epithelial tolerance breakdown are pathways that might help to clarify the potential contribution of the microbiota to SS pathogenesis. This review aims to provide an overview of recent studies describing microbiota changes and microbiota mechanisms associated with Sjögren’s syndrome. Data on the microbiota in SS from PubMed, Web of Science, Scopus and the Cochrane Library databases are summarized. Overall, the microbiota makes a major contribution to the development of Sjögren’s syndrome and progression. Future microbiota studies should improve the management of this heterogeneous autoimmune disease.

## 1. Introduction

Primary Sjögren’s syndrome (pSS) is a heterogeneous chronic autoimmune disorder with multiple clinical manifestations that can develop into non-Hodgkin’s lymphoma in mucosa-associated lymphoid tissue (NHLMALT) [1]. The pathogenesis of Sjögren’s syndrome (SS) is not completely understood, although it is assumed that pathogenesis of SS is multifactorial. However, no causal association currently exists that might explain the aberrant immune response targeting multiple epithelial structures that in turn give pSS its characteristic presentation [1,2,3,4,5].

The notable contribution of the microbiome to the development of autoimmune disorders was demonstrated by the Human Microbiome Project [6]. The highest density and diversity of microbes is concentrated in the gastrointestinal tract, though microbes are present on all body surfaces that are exposed to environmental factors, including the respiratory tract, mouth, reproductive and urinary tracts and skin. It is now clear that the microbiota both locally and systemically influences the development and functioning of all mammalian body systems. Human commensal microbes maintain developmental homeostasis in many systems and regulate the development of specific tissue cells in those systems [7]. As microbiota research continues to grow, its role in autoimmunity is becoming increasingly apparent. Dietary and probiotic therapy, as well as fecal microbiota transplantation, have beneficial effects in the management of certain autoimmune disorders [8,9]. Some autoimmune rheumatic diseases, such as rheumatoid arthritis and systemic lupus erythematosus, have been shown to be characterized by a dysbiosis of the intestinal microbiota. It should be mentioned that, thanks to the latest technologies, evidence has emerged that dysbiosis of the skin microbiota as one of the main features of the onset and progression of atopic dermatitis opens up new avenues for the development of therapies for this disease. Probiotic and prebiotic preparations, and even transplantation of the skin microbiota, are finding their way into clinical application and promise excellent avenues for the management of atopic dermatitis [10,11].

There is a growing body of research studies on alterations in the diversity of the microbial communities of the intestinal, oral and ocular regions and the associations of dysbiosis with disease activity and expression of symptoms in pSS patients [6,12].

This review aims to provide an overview of recent studies describing microbiota changes and microbiota mechanisms associated with Sjögren’s syndrome. Data on the microbiota in SS from PubMed, Web of Science, Scopus and the Cochrane Library databases are summarized.

## 2. The Interaction between Human Microbiota and Immune System

Approximately 10–100 trillion symbiotic microorganisms form communities and are present in every habitat of the body, such as the mouth, respiratory tract, gastrointestinal tract, reproductive and urinary tracts and skin, and they make up the human microbiota [13]. Homeostatic and changing interactions are common between microbes and the host. Through host–microbiota coevolution, the microbiota supports the adaptation and maintenance of human health. The elimination of harmful pathogens and the selective promotion of commensal tolerance are due to the interaction between members of the innate and adaptive immune systems. Pathological conditions in the body, including autoimmune rheumatic diseases, can result from the influence of environmental or genetic factors on the interaction between the microbiota and the immune system [14,15,16].

Exposure to microbes early in infancy is relevant for the development of the immune system. Breastfeeding is important for the formation of the early gut microbiota and for the stimulation of the infant immune system, as bacteria and parts of bacteria from the gastrointestinal tract enter the mammary gland [17]. Microbial metabolites help to maintain the balance of the intestinal mucosa. For example, indole-3-aldehyde produced by *Lactobacilli* induces the production of interleukin 22 (IL-22) and interleukin 17 (IL-17), which activates the aryl hydrocarbon receptor. Symbiosis of the gut microbiota is essential for the development of gut-associated lymphoid tissues [18]. Changes in the microbiota can disrupt functions of the immune system. This has been confirmed by studies in animal models, which have shown that the microbiota maintains a balance between the host’s immune system and microbes [19]. The bidirectional relationship between the microbiota and innate immunity is due to the activation of specific signaling molecules produced by host and gut bacteria, culminating in monocytes, macrophages and innate lymphoid cells that form the intestinal endothelial barrier. The link between the microbiota and tissue-dwelling dendritic cells, which is crucial for the development of immune memory and tolerance, should be emphasized. Recently, mechanisms regulating the reciprocal relationship between the microbiome and the adaptive immune system have been uncovered, in parallel to their influence on innate immune function [20]. The interface between gut microbiota and B cells promotes the proliferation of Forkhead box P3 (Foxp3+) regulatory T cells (Tregs). The interaction of the gut microbiota with gut regulatory CD4+ T cells maintains intestinal balance. The adoptive T cell response is regulated by the microbiome via CD8+ (cytotoxic) T cells, which are able to eradicate intracellular pathogens and cancer cells. It is important to emphasize that the interface between intestinal bacteria and T follicular helper (T fh) cells, which are B cell helper cells, creates a balance in the microbiota [21].

## 3. Factors Contributing to Microbiota Dysbiosis

**Figure 1:**
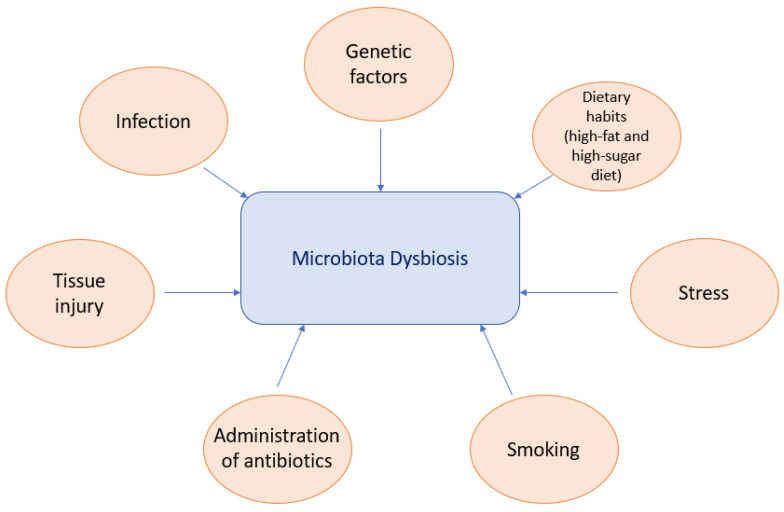
Driving factors of microbiota dysbiosis (Huang, X.; et al., 2023) [22].

Microbiota dysbiosis has a major impact on the onset and development of numerous systemic diseases. In individuals with a genetic predisposition, a variety of factors such as diet, residence place changes, environmental modification or antibiotic use can disturb the microbiome, disrupt the host–microbiome interface or change the immune function, leading to an altered immune response (Figure 1) [21,22].

Obesity has a negative impact on the development of type 1 diabetes, inflammatory bowel disease and a number of autoimmune diseases. The intake of carbohydrate-rich foods and industrially processed foods, as well as sugar, is a major dietary change. Eating high amounts of fat and sugar triggers neural triggers that signal a lack of food. This is due to an increase in levels of the neurotransmitter dopamine, which regulates the experience of pleasure. In addition, high fat and sugar intake can lead to dysbiosis of the gut microbiota, which in turn can lead to a variety of disorders, including non-alcoholic fatty liver disease. Diet has been reported to be a central contributor to altering the balance of commensal microbiota, as it was demonstrated that some mouth pathogens were more prevalent in hunter–gatherers than in conventional farmers [23,24,25]. Dietary fat suppresses systemic inflammation and decreases plasma Tumor necrosis factor α (TNF-α) levels in animal models [26]. In addition, in animal models, high-fat diets have been shown to alter oral microbiota and neutrophil enrichment [27]. Lipid metabolism in the liver can be affected by the use of large amounts of sugar. This changes the composition of gut microbes and can lead to hyperlipidemia [28]. A healthy diet, with probiotics or prebiotics, is able to correct the imbalance of the microbiota when it is disturbed by an unhealthy diet [29,30]. Irrational use of antibiotics disturbs the equilibrium of the gut microbiota and enhances antibiotic resistance by reducing the body’s resistance to pathogenic microorganisms. The reduction in the diversity of the gut microbiota with antibiotic treatment has been shown in animal models [22,31]. Tissue damage causes damaged cells to produce chemical signals that promote the dissemination of pathogenic microorganisms [32]. *Porphyromonas gingivalis* has the ability to affect the complement system mechanism, which can lead to microbiota dysbiosis [33]. *Porphyromonas gingivalis* induces the expression of phospholipase A2-IIA (PLA2-IIA) in oral epithelial cells, which is related to microbiota dysbiosis in the mouth [34]. The gut and oral microbiome can be altered by psychological stress. Recent evidence suggests that there is a microbiota–oral–gut–brain axis that operates during psychological stress [35].

## 4. Microbiota in Sjögren’s Syndrome

There are more than 80 autoimmune diseases that are common in humans. A combination of genetic and environmental factors acting on the immune system leads to an immune response to these factors and the development of autoimmune diseases. The immune response to self-antigens, development of abnormal autoantibody-producing B cells, formation of autoreactive T cells and secretion of pro-inflammatory cytokines, all play a role in the development of autoimmune diseases. Evidence is mounting that microbiota dysbiosis is an influential factor in the causation of autoimmune diseases [32]. An imbalance of gut microbes is capable of inducing an increase in interferon-alfa (IFN-α) and interleukin 33 (IL-33) production by plasmacytoid dendritic cells and of triggering autoimmune pancreatitis [36]. Studies of experimental autoimmune encephalomyelitis in animal models have shown that dysbiosis of the gut microbiota leads to an increase in Th17 and effector Th17 (Tef17) cells in both the brain and spinal cord, as well as inducing the secretion of serum cytokines [37]. Understanding the link between microbiota and autoimmunity is essential to improve the management of autoimmune diseases [32].

### 4.1. Effects of Microbiota on Sjögren’s Syndrome Pathogenesis

The microbiota makes a marked contribution to the development of Sjögren’s syndrome. Although it has been said that the causality between them is unknown, this has been overturned by a recently published study. This study provides evidence for causal effects of the structure of the intestinal microbiota and associated genes in relation to the risk of SS: the family *Porphyromonadaceae*, genus *Subdoligranulum*, genus *Butyricicoccus* and genus *Lachnospiraceae* are related to a lower likelihood of SS, and the genus *Fusicatenibacter* and genus *Ruminiclostridium9,* in contrast, are related to a higher likelihood of SS [38].

Gut dysbiosis causes an imbalance in the immune system, which can lead to the development or worsening of autoimmune disorders, including rheumatic autoimmune diseases [39,40]. The diversity of the gut microbiota is significantly reduced and changed in patients with SS when compared to healthy controls [41]. The intestinal microbiota diversity and seriousness of dry eye symptoms are significantly correlated in patients with SS [42]. Frequently, the ratio of *Firmicutes*/*Bacteroidetes* (F/B) is found to be lower in patients with autoimmune diseases, and it can be a mark of gut microbiota dysbiosis [43,44,45]. Studies on the contribution of the microbiota to the development of SS suffer from major limitations due to differences in the ethnic origin, gender or diet between studies’ cohorts, small sample sizes, and different sequencing methods, leading to a wide range of study results.

**Figure 2:**
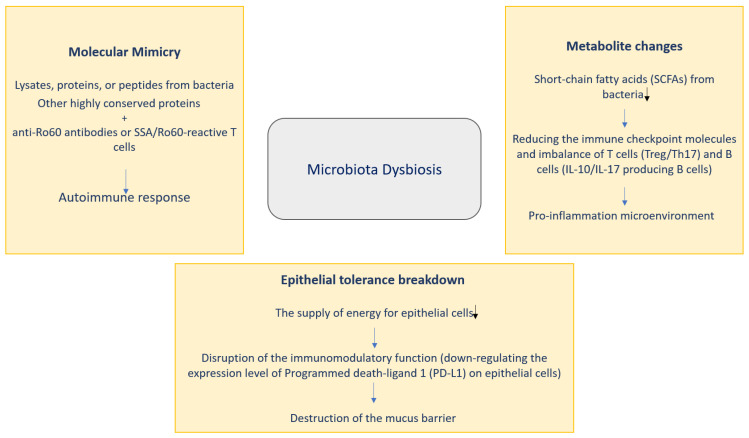
Potential role of microbiota in SS pathogenesis.

Three pathways might help to clarify the potential contribution of microbiota to SS pathogenesis: molecular mimicry, metabolite changes and epithelial tolerance breakdown (Figure 2).

#### 4.1.1. Molecular Mimicry

Molecular mimicry is a part of the process of autoimmunity induced by infectious factors with intermolecular spread of epitopes. B cell crosstalk between the *Coxsackie* virus 2B protein and Ro60 has an important link to the generation and survival of the response of anti-Ro60 autoantibodies, and this anti-Ro60 autoantibody response is necessary for SS pathology [6]. It has been shown that serum from patients with positive anti-Ro60 is reactive with *Bacteroides thetaiotaomicron* lysates [46]. A novel antigen called the outer membrane protein (*OmpA*) from *Escherichia coli* is very antigenic, inducing antibodies to SSA/Ro and SSB/La and causing Harderian and salivary gland inflammation [47], suggesting that molecules of microbial origin specifically induce inflammation in exocrine glands. The von Willebrand factor type A domain protein (vWFA), derived from *Capnocytophaga ochracea*, demonstrates the most potential to activate SSA/Ro60 reactive T cells, leading to produce IL-2 at high levels. Furthermore, whole *Escherichia coli* expressing vWFA and recombinant vWFA protein may initiate Ro60-reactive T cell activation [6]. Microbial proteins from *Staphylococcus aureus* and *Escherichia coli* can result in the production of appropriate antibodies in SS patients, and due to molecular mimicry, patients with SS may develop fatigue [48]. These findings support the contribution of molecular mimicry as a possible mechanism of action in SS pathology.

#### 4.1.2. Metabolite Changes

Metabolite changes are thought to be a key factor in the pathogenesis of SS. Changes in the metabolites of short-chain fatty acids (SCFAs) are some of the most relevant in gut microbes’ metabolites. SCFAs monitor the immune system and host metabolism [6]. It is important to note that SCFA-producing bacteria have significant pro-regulatory and tolerogenic effects on the immune system. It has been observed that SCFA-producing bacteria such as *Lachnoclostridium*, *Lachnospira* and *Sutterella* are reduced in patients with autoimmune diseases. [49]. Recently, the bacterial product butyrate has attracted considerable interest in SS. Evidence has already shown that bacteria that produce butyrate, such as *Faecalibacterium prausnitzii*, *Bacteroides fragilis*, *Lachnoclostridium*, *Roseburia*, *Lachnospira* and *Ruminococcus*, are greatly reduced in patients with SS [50,51,52]. Butyrate maintains intestinal barrier processes by providing energy to colonic epithelial cells. Some studies have shown that the butyrate-producing bacteria *Bacteroides* spp., *Clostridia clusters XIVa* and *IV* were essential for maintaining the Treg/Th17 ratio. Importantly, the polysaccharide as an immunomodulatory molecule acts in combination with butyrate to promote Treg cell formation [6]. A disturbance in the Treg/Th17 ratio impairs the ability of the mucosal barrier to prevent colonization by harmful pathogens. [49]. The point is that butyrate regulates genes related to the circadian rhythm to exert an anti-inflammatory function, which may alter the balance of T cells and monitor the level of B cells producing interleukin 10 (IL-10) and interleukin 17 (IL-17) [6]. On the other hand, butyrate can increase the rate of salivary secretion and ease inflammation of the salivary glands [6]. All this suggests that decreased SCFA or bacteria that produce butyrate may modulate the mucosal barrier and functions of immune system [6,53].

#### 4.1.3. Epithelial Tolerance Breakdown

Several studies have reported that salivary gland epithelial cells (SGECs) may affect the immune response, possibly due to bacterial involvement [5]. *Haemophilus parainfluenzae* has an immunomodulatory function as a commensal bacterium in patients with SS. Downregulation of *Haemophilus parainfluenzae* may reduce programmed death ligand 1 (PD-L1) on SGECs and inhibit control of the proliferation of CD4+T. Salivary glands of SS patients were observed to be infected with bacteria [54,55]. The oral bacterium *Prevotella* is associated with SS. This bacterium can modulate major histocompatibility complex (MHC) molecules and CD80 submandibular gland tumor cells in humans, which can lead to inflammation induced by interferon (IFN) [50]. Considerably higher prevalence of *Prevotella* has been found in a family containing key mucin-degrading enzymes that can also impact on the gut mucus [56]. Apart from lowering the energy intake, impairing the immunomodulatory activity of epithelial cells and disrupting the mucus barrier, there are other potential pathways for the microbiota to reach the degradation target in Sjögren’s syndrome. Correlation between bacterial perturbations and pathological alterations has been noted; the causal relationship needs further investigation. *Staphylococcus aureus* and coagulase-negative *Staphylococci* sp. Species have been shown to be markedly elevated and correlated with elevated infiltration with neutrophils into the conjunctiva in trombospondin 1 decient (TSP-1-/-) mice [57]. Crucially, molecular mimicry, changes in metabolites and epithelial tolerance breakdown are not totally autonomous, but may interfere with one another, making the analysis of microbiota causality for SS challenging.

### 4.2. Characteristic Changes in Microbiota in Patients with Sjögren’s Syndrome

A growing body of research shows that lower microbiota diversity can lead to higher disease activity in patents with SS. An early study found that treatment with oral antibiotics and desiccation caused extensive changes in the gut microbiota [6]. Fecal microbiota diversity was adversely correlated with the combined eye and systemic disease index. In addition, the European League Against Rheumatism (EULAR) Sjögren’s syndrome disease activity index (ESSDAI) total score, Clinical European League Against Rheumatism Sjögren’s Syndrome Disease Activity Index (ClinESSDAI) total score and the fecal calprotectin level were all higher, and the complement component 4 level was lower, in SS patients with severe dysbiosis [58,59]. In addition to these findings, a significant positive correlation was observed between tear break-up time and *Actinobacteria* and *Bifidobacteria.* Also, a recent study has indicated marked changes in the *phyla* and *genera* in patients with SS and non-SS in comparison to healthy control and suggested a probable correlation of prevalent pro-inflammatory bacteria with SS and non-SS [50,60]. A positive correlation between the levels of the pro-inflammatory cytokines interleukin 6 (IL-6), interleukin 12 (IL-12), interleukin 17 (IL-17) and TNF-α and *Enterobacter* abundance was observed, but these cytokines were negatively correlated with the abundance of *Bifidobacterium, Blautia, Lachnospira*, *Roseburia* and *Ruminococcus* in SS patients. In one study, a strong inverse correlation was found between *Parabacteroides distasonis* abundance and IL-6 and TNF-α level in the control group in comparison to SS patients [51]. A summary of the latest studies on the characteristic changes in the microbiota in SS published this year is presented in the table below (Table 1).

The gut–ocular–oral axis is an outstanding representation of the contribution of the microbiota to the development of SS. Transplantation of feces from normal mice into CD25 knockout mice can modify the pathology in the gut, ocular and oral region [55]. Consequently, patients with SS may have a gut–eye–mouth axis. When considering the ocular–gut axis, studies demonstrated that intestinal dysbiosis is also associated with the seriousness of ocular mucosal disease in patients with SS. The relative presence of *Bacteroidetes*, *Actinobacteria* and *Bifidobacterium* in the intestines of SS patients was markedly correlated with symptoms of dry eye [50]. Pretreating with desiccation and antibiotics demonstrated pronounced changes in the gut microbiota, partly due to *Proteobacteria,* that were correlated with a more severe eye phenotype in animal models [59]. These data indicate that gut dysbiosis has an important clinical relationship with the intensity of dry eye symptoms. It is hypothesized that gut microbiota dysbiosis may exacerbate the loss of the germinal center in dry eye by altering natural killer/natural killer T (NK/NKT) cells and cytokine profile, which may lead to impaired Th2 [59]. In one study, the *Firmicutes*, *Actinobacteria*, *Proteobacteria* and *Bacteroidetes* phyla were identified as predominant in the conjunctiva from SS patients and the control group [59]. There was no significant difference in the variety and content of the eye surface microbiome in SS patients and controls. In addition, eye and oral axis tests are scarce, and no microbiota-associated results were observed. It should be mentioned that intercellular bacteria living in the buccal epithelium can be transferred to the intestine via the process of buccal epithelial cell loosening [6]. The proportionate presence of *Actinomyces* and *Lactobacillus* in samples from the mouth was related to their presence in fecal samples from patients with SS [64]. A recent study focused on differences in intestinal microbiota between pSS patients and healthy controls at the genus and species levels, and this enables a more precise understanding of the role of the microbiota in the pathogenesis of pSS. In addition, certain genera and species were associated with the seriousness and treatment defiance of pSS. Some genera (*Escherichia-Shigella*, *Insteinimonas*, *Lactobacillales*, *Sardovia*, *Veillonella*) and species (*Escherichia coli*, *Fusobacterium ulcerans Lactobacillus* phage Sal3, *Lactobacillus reuteri*, *Lactobacillus gasseri*, *Streptococcus lutetiensis*, *Streptococcus mutans*, *Scardovia wiggsiae*) have been found in higher levels in the feces of pSS patients than in that of healthy controls. *Escherichia coli* in pSS patients was found to have an amino acid sequence similar to that of pSS-relevant autoepitopes. This suggests that *Escherichia coli* contributes to the pathogenesis of pSS [65]. Recently, thanks to the results of studies in mice, it has been suggested that Lactobacillus acidophilus and propionate have therapeutic potential for the treatment of SS. Dysbiosis of the intestinal microbiota induces SS by enhancing lymphocyte infiltration into the salivary glands and by stimulating stromal interaction molecule type 1—the interferon gene stimulator-type I interferon axis (STIM1-STING-IFN axis). These results provide support for the idea that *Lactobacillus acidophilus* and propionate have a therapeutic potential for the treatment of SS [66]. Several studies have shown that commensal oral and intestinal bacteria are cross-reactive with SSA/Ro60, and this is critical for the development of SS. Much of the genetic material produced by commensal microbiota encodes hypothetical proteins whose functions are currently unknown, sometimes known as genetic dark matter. Some microbial products with intriguing and profound implications for host biology have been characterized from previously undescribed genes. The high-throughput zebrafish model has been shown to be particularly useful for the extraction of microbial dark matter by revealing the secreted bacterial proteins that modulate neutrophil activity and β cell proliferation, respectively. Through further investigation of this new biological source material, many new modulators of host health will undoubtedly be discovered [6]. There is likely to be a connection between oral and intestinal bacteria, although the principle of action is not clear. In the future, it will be important to investigate the interconnectedness of the microbiota in different parts of the body in patients with Sjögren’s syndrome, including those body systems characterized by autoimmune epithelitis and xerosis.

## 5. Conclusions

Overall, studies have shown that the microbiota of patients with Sjögren’s syndrome makes a major contribution to the development of the disease and its expression and progression. Future microbiota studies should improve the management of this heterogeneous autoimmune disease.

## Figures and Tables

**Table 1:** The latest studies on the characteristic changes in the microbiota in SS patients.

Reference	Sample	Case and Control	Conclusion of the Study
[38]	Gut microbiome	-	Family *Porphyromonadaceae*, genus *Subdoligranulum*, genus *Butyricicoccus* and genus *Lachnospiraceae* are associated with a lower chance of SS developing. Genus *Fusicatenibacter* and genus *Ruminiclostridium9* increase chance of SS developing.
[60]	Tear film	- SS (*n* = 17) - Non-SS (*n* = 28) - Healthy (*n* = 33)	Changes in the *phyla* and *genera* in SS and non-SS are significant (compared with healthy controls). An expected relation of prevalent pro-inflammatory bacteria with SS and non-SS.
[61]	Fecal, oral and vaginal samples	- Healthy (*n* = 40) - pSS (*n* =133) - Non-pSS (*n* = 56)	Microbial shifts could appear prior to pSS.
[62]	Saliva	- SS with candidiasis (*n* = 5) (decayed, missing and filled teeth (DMFT) score 22) - Patients with oral candidiasis (*n* = 5) (DMFT score 17) - Healthy with active caries (*n* = 5) (DMFT score 14)	The primary genera for all: *Treponema, Lactobacillus, Streptococcus, Selenomonas and Veillonella*. The most abundant significantly mutative taxonomy (I001): *Veillonella parvula*. Microbial diversity significantly increased in SS. Different microbial compositional heterogeneity in SS. Microbial dysbiosis differs in SS independent of oral *Candida* carriage and DMFT.
[63]	Fecal samples	- Anxiety (*n* = 17) - Non-anxiety (*n* = 39) - Patients with dry eye due to pSS (*n* = 56)	Correlation between anxiety disorders and intestinal dysbiosis. *Prevotella* is associated with seriousness of dry eye.
[64]	Single-center, prospective, comparative, randomized, double-blind, cross-over controlled study to assess the tolerance and effectiveness of adhesive biofilms	- pSS (*n* = 10) with hyposialia	The sodium alginate biofilm increased the abundance of the *Treponema genus* in mouth. The prebiotic biofilm increased the abundance of the genera *Veillonella* and *Prevotella*. Pre-treatment with the prebiotic biofilm prevented the emergence of the *Treponema* genus induced by subsequent treatment with the sodium alginate biofilm. Pre-treatment with the prebiotic biofilm has a potential protective effect.
[65]	Fecal samples	- pSS (*n* = 30) - Healthy (*n* = 30)	The composition and function of the intestinal microbiota are common in pSS patients. Certain genera and species are associated with seriousness and therapy defiance of pSS disease.

## Data Availability

Publicly available datasets were analyzed in this study and referred to in the list of references.
